# Appendiceal Abscess Within a Giant Amyand’s Hernia: A Case Report

**DOI:** 10.7759/cureus.36947

**Published:** 2023-03-31

**Authors:** Si Louise Sun, Kerry L Chen, Chahaya Gauci

**Affiliations:** 1 Department of Surgery, Hepatobiliary and Surgical Oncology Unit, St. George Hospital, Kogarah, AUS; 2 Department of Surgery, Faculty of Medicine, The University of New South Wales, Sydney, AUS

**Keywords:** giant amyand's hernia, appendiceal abscess, amyand’s hernia, giant inguinoscrotal hernia, appendicitis

## Abstract

Amyand’s hernia is a rare clinical entity, defined as an inguinal hernia containing the appendix. Giant inguinoscrotal hernia is also a rare clinical finding that presents major operative dilemmas due to the loss of abdominal domain. Here, we report a case of a 57-year-old male who presented with a giant irreducible right inguinoscrotal hernia and obstructive symptoms. The patient underwent an emergency open right inguinal hernia repair, where an Amyand’s hernia was identified. The hernia contained an inflamed appendix and associated abscess, caecum, terminal ileum, and descending colon. Using the giant sac to isolate the contamination, an appendicectomy was performed, the hernial contents reduced and the hernia repair reinforced with partially absorbable mesh. The patient recovered post-operatively and was discharged home with no recurrence on four-week follow-up. This case provides learning points on decision-making and surgical management of a giant inguinoscrotal hernia containing an appendiceal abscess, also known as Amyand's.

## Introduction

Inguinal hernias are a common general surgical presentation, accounting for 75% of abdominal wall hernias. Amyand’s hernias are rare inguinal hernias that contain the appendix, present in 1% of cases [1]. They may be associated with appendicitis, abscess formation, or appendiceal perforation, resulting in sepsis and significant mortality (15-30%) [2]. Giant inguinoscrotal hernias are defined as those extending below the midpoint of the inner thigh on standing and account for 2.8-5% of inguinal hernias. They are associated with an increased risk of abdominal compartment syndrome upon reduction and repair [2,3]. There is limited literature on Amyand’s hernias, with less than ten case reports of giant Amyand’s hernias. We describe a case of giant Amyand’s hernia containing an appendiceal abscess.

## Case presentation

**Figure 1:**
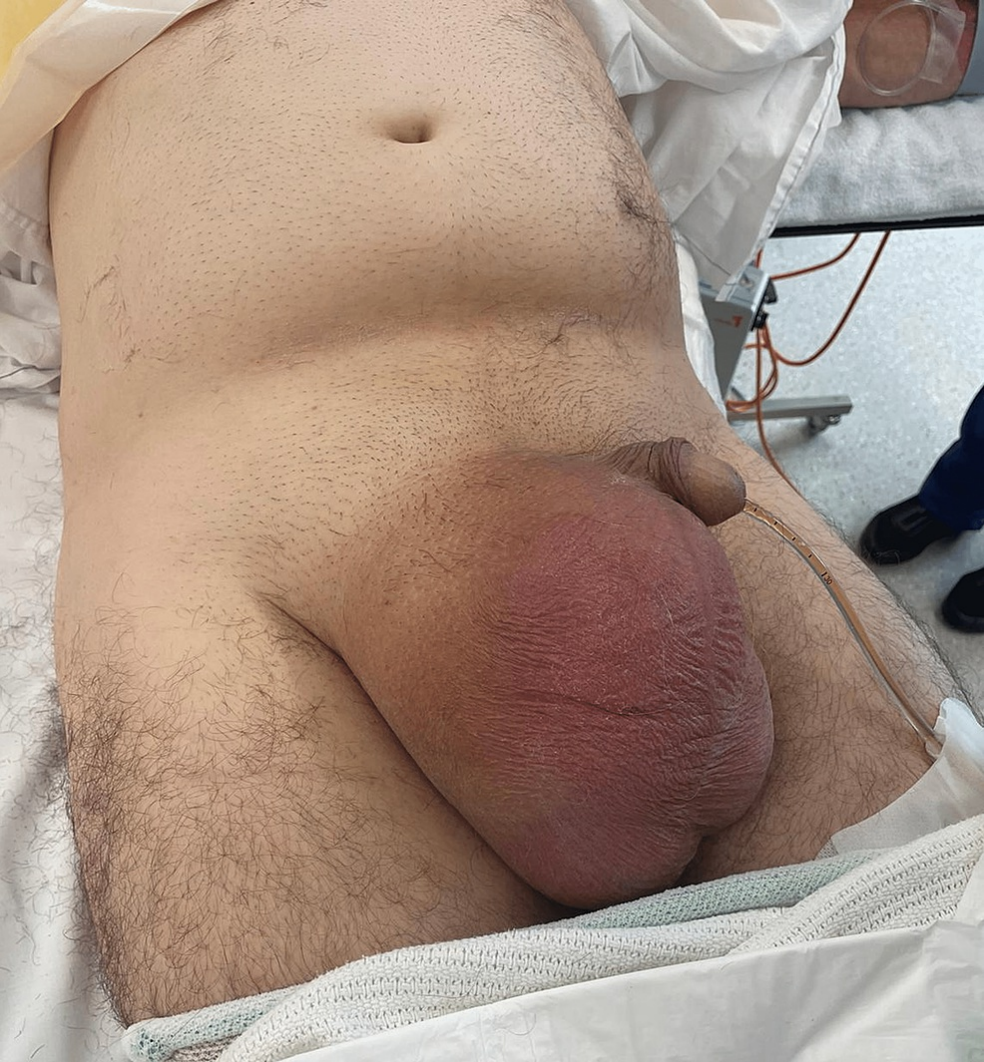
Giant right irreducible inguinoscrotal hernia (supine position)

A 57-year-old man presented to our institution with an irreducible, giant right inguinoscrotal hernia which had been previously reducible and slowly enlarging over many years. This was associated with pain, nausea, and vomiting. He was an ex-smoker of 10 pack-years, but had no other relevant medical or surgical history. On examination, he was hemodynamically stable and afebrile. The abdomen was soft, mildly distended, with no tenderness on palpation. A giant right inguinoscrotal hernia was evident, which extended below the midpoint of his inner thigh (Figure 1).

The overlying skin was tender, firm, and erythematous. Blood tests showed a raised c-reactive protein of 300 mg/L with a normal white cell count of 9.8x10^9^/L, and an acute renal injury with creatinine of 177 µmol/L and estimated glomerular filtration rate of 36 mL/min/1.73 m^2^.

**Figure 2:**
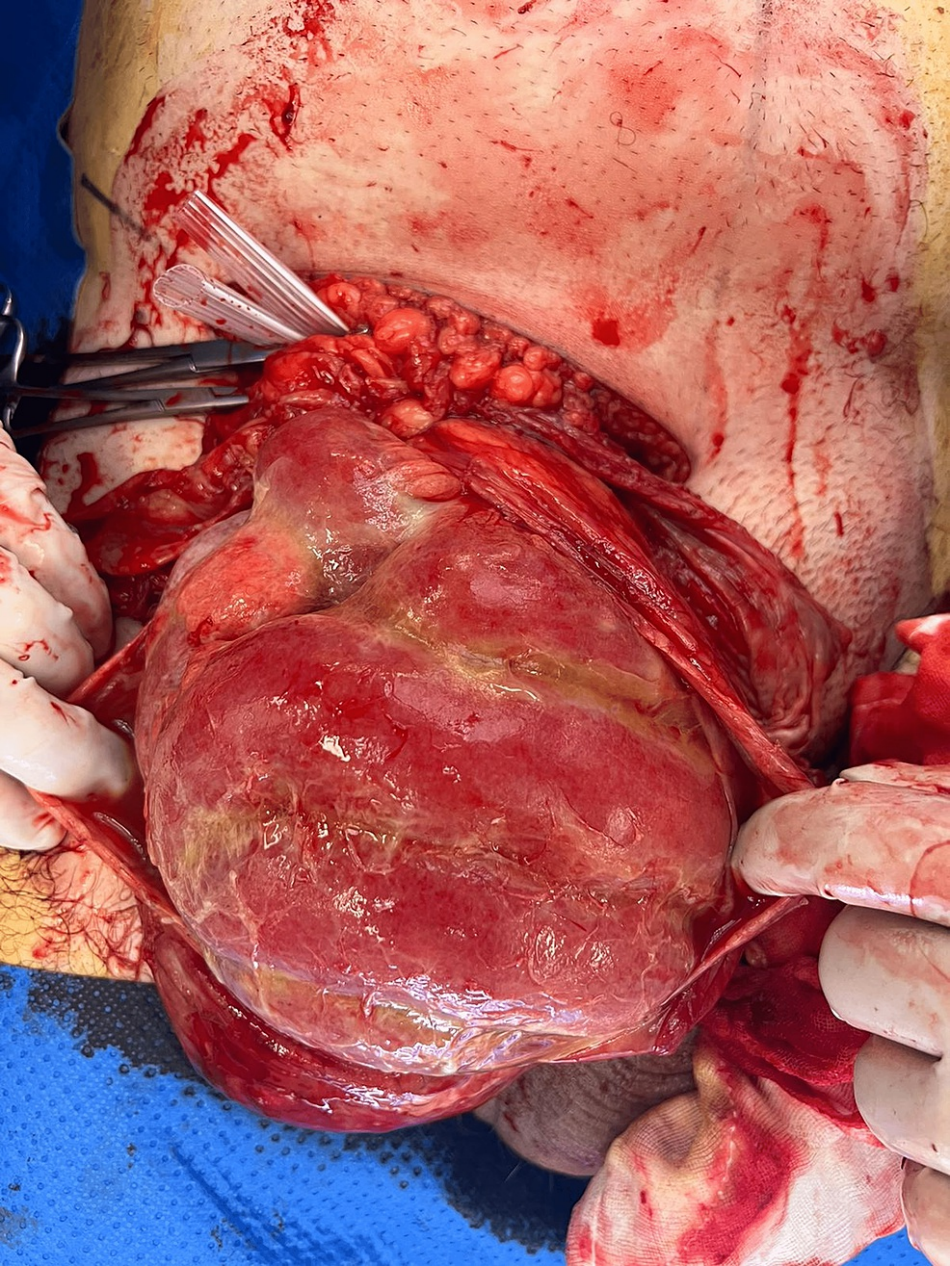
Hernia sac containing suppurative appendicitis, terminal ileum, cecum, and segment of descending colon

**Figure 3:**
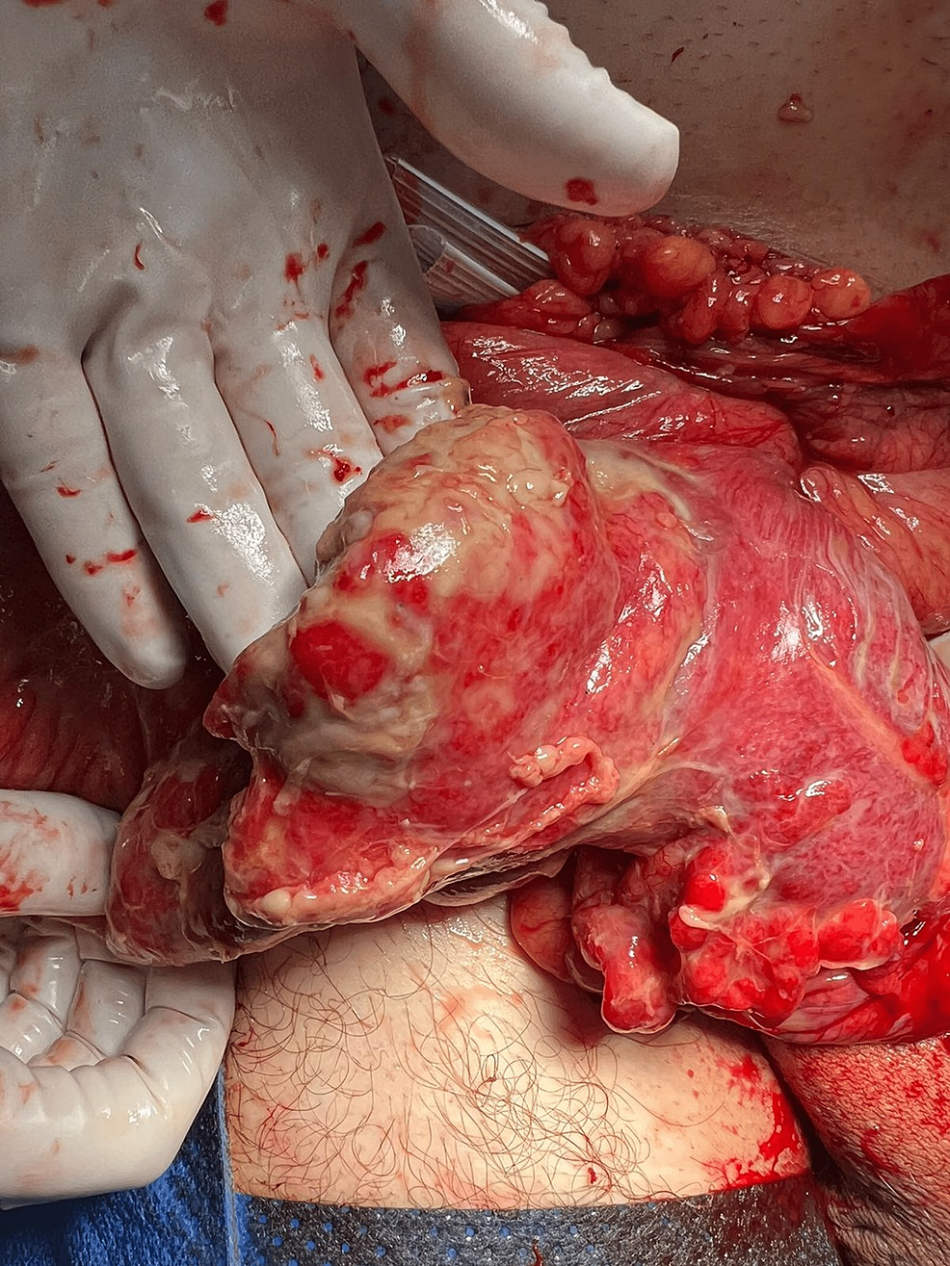
Acute appendicitis and contained abscess within the hernia sac

Given the clinical concern for bowel obstruction and ischemia, this patient proceeded to urgent operative management without imaging. He underwent an open right hernia repair via Lichtenstein approach. The hernia sac revealed acute appendicitis and a contained abscess, along with fibrinous adhesions to the cecum, terminal ileum, proximal descending colon, and bladder edge (Figures 2, 3).

The wound was protected and an appendicectomy was performed with a gastrointestinal anastomosis 80 mm stapler. The staple line was oversewn with 3-0 vicryl and the remainder of the hernial contents were inspected to ensure there was no additional pathology. The giant hernial sac and an Alexis wound retractor were used to contain any contamination as 3 L of saline wash was applied. This was performed until clear returns were seen. Next, the excess hernia sac was excised, and the remainder closed with 2-0 vicryl. The posterior wall of the inguinal canal was plicated with 2-0 vicryl, and the repair was reinforced with ULTRAPRO® partially absorbable mesh (Ethicon Inc., Somerville, NJ). The external oblique aponeurosis and subcutaneous space were closed with 2-0 vicryl. The right testicle was confirmed viable, and a 15 Fr Blake® drain (Ethicon Inc., Somerville, NJ) was inserted into the right scrotum. Local anesthetic was infiltrated and the skin was closed with staples.

The patient was admitted to the Intensive Care Unit (ICU) post-operatively due to oligoanuric renal failure. This was likely due to acute tubular necrosis, secondary to obstruction and hypovolaemia. His renal function improved post-operatively with additional fluid rehydration. He was stepped down from ICU on day two post-operatively, and completed five days of intravenous ceftriaxone and metronidazole before being discharged home on oral antibiotics, as per infectious diseases advice. The right scrotal drain remained in situ on discharge to prevent post-operative seroma formation. The patient was also educated on compliance with scrotal support underwear and to avoid heavy lifting or straining.

Histopathology of the appendix confirmed acute appendicitis, with transmural inflammatory infiltrate and a meso-appendiceal abscess. The patient recovered well, with improvement in renal function on one-week follow-up. His drain and skin staples were removed uneventfully following this. On four-week follow-up, he remained well with no hernia recurrence.

## Discussion

Amyand’s hernia is a rare clinical finding, present in 1% of all inguinal hernias. Clinical presentation varies from generalized abdominal pain, localized right lower quadrant tenderness to irreducible groin mass or bowel obstruction [1,4].

The presence of appendicitis within an Amyand’s hernia is an even rarer complication, estimated to occur in 0.07-0.13% of all inguinal hernias [4]. Other complications include abscess formation, perforation, and necrotizing soft tissue infection [5]. The pathophysiology of acute appendicitis in Amyand’s hernia is thought to be related to adhesional incarceration of the appendix within the hernia, with compression of the external inguinal ring resulting in luminal obstruction, bacterial overgrowth, and transmural inflammation [6].

Due to the range of clinical presentations, Amyand’s hernias are difficult to diagnose pre-operatively without imaging. The majority are diagnosed intraoperatively, which may complicate operative planning, particularly in the presence of unexpected infection or inflammation.

There is no gold standard or expert consensus on the management of Amyand’s hernias. Lossannof and Basson suggest four classifications of Amyand’s hernias based on appendiceal pathology which may guide management [7]. Type 1 describes the presence of a normal appendix and may be managed with tension-free mesh hernia repair. Generally, appendicectomy is not recommended due to infection risk in an otherwise clean operation [3]. Type 2 Amyand’s hernias are those with acute non-perforated appendicitis. Suggested management involves appendicectomy with endogenous hernia repair. Mesh traditionally is not used due to the risk of wound infection and breakdown [6]. However, there is a growing body of literature that suggests hernia repair with synthetic mesh is acceptable in select patients with clean-contaminated and contaminated fields [8,9]. Type 3 involves acute appendicitis with perforation and evidence of intra-abdominal sepsis and warrants laparotomy, appendicectomy, washout, and primary endogenous hernia repair. Lastly, type 4 is classified as acute appendicitis complicated by other intra-abdominal pathology, including incidental masses or tumors. Management should be as per types 1-3 with further investigation of the second pathology.

This patient had a type 2 Amyand’s hernia. While ideally mesh would be avoided, there was the complicating factor of a giant inguinoscrotal hernia. The use of synthetic mesh was therefore favored to reduce risk of hernia recurrence given the defect size [10]. As the suppurative process involving the appendix was separated from the wound and mesh site and adequate washout was achieved, the decision was made to use ULTRAPRO® mesh (Ethicon Inc., Somerville, NJ), a partially absorbable poliglecaprone/polypropylene mesh combined with intravenous antibiotic coverage. Staged repair was considered less ideal due to the increased cost, difficulty in achieving adequate anatomical planes, and risk of patient non-compliance and loss to follow-up. It was evident on immediate one-week and four-week follow-up that this was a successful repair in terms of both appendicitis and hernia management.

## Conclusions

This study on the repair of a rare giant type 2 Amyand’s hernia adds to the literature on operative options for Amyand’s hernia. Surgeons should be aware of the possibility of intra-operative diagnosis of Amyand’s and the surgical considerations for management. There is growing evidence to support the efficacy of synthetic mesh repair compared to endogenous repair in contaminated fields, with comparable rates of surgical site infection. However, further high-quality studies with long-term follow-up are warranted to establish a consensus. Given the rarity of such cases, we recommend taking a case-by-case approach to allow for both optimal hernia repair as well as sepsis control.
